# Detachable Lip and Cheek Plumper for Rehabilitation of Facial Disfigurement

**DOI:** 10.1155/2021/6668737

**Published:** 2021-05-13

**Authors:** Nada Fathalla Abdelbagi, Ibrahim Ahmed Ismail, Fadia Awadalkreem, Mohammed Nasser Alhajj

**Affiliations:** ^1^Department of Oral Rehabilitation, Faculty of Dentistry, University of Khartoum, Khartoum, Sudan; ^2^Department of Prosthodontics, Faculty of Dentistry, Thamar University, Dhamar, Yemen

## Abstract

**Background:**

Hemimaxillectomy of acquired palatal defects may predispose the patient to hypernasal speech, leakage of fluids into the nasal cavity, impaired masticatory function, and swallowing difficulties leading to a detrimental impact on the quality of life. Sequentially, it can also affect individual character and trust in social life, leading to social phobia and anxiety. This article presents prosthodontic management of a young male patient with deformation of the entire right half of the face due to surgical removal of odontogenic myxoma. It describes a method where the clinician utilized a simple, noninvasive, and cost-effective technique to cut the weight of the obturator and to attain aesthetics, utilizing a detachable lip and cheek plumper. *Case Presentation*. A 19-year-old male attended the Prosthodontic Clinics at Faculty of Dentistry, the University of Khartoum, eight months after the surgical removal of a tumor. The chief complaint was difficulty eating or drinking. Furthermore, he dropped out of school because of his facial deformity and his indistinct voice. Medical and dental history revealed surgical resection of the entire right half of the maxillary alveolar bone due to myxoma. Intraoral examination revealed a well-healed surgical defect in the maxillary right buccal vestibule creating an oroantral communication. A treatment plan was formulated, keeping the patient's demand in mind. The decision was made to treat the patient with a maxillary obturator that would gain its support from the remaining teeth and tissues with a detachable cheek and lip plumper. The use of dental magnets as a means of attachment was elected.

**Conclusion:**

This article has described a simple, noninvasive, and cost-effective method to improve facial appearance in patients with hollow faces. The plumper prosthesis successfully improved the patient's appearance to the extent that comfort and function would permit and encourage self-esteem.

## 1. Background

The face is a significant constituent of body image; and those with facial disfigurement, self-worth, and self-concept are tightly entangled. It carries on how one is perceived and judged by others, guiding their impressions and behavior [1]. Most body deformities can be obscured from sight, but those of the face are open for all to view [2]. A young person might hold exterior looks to be very important; contrary to our thought, disfigurement to females is equally distressing to male patients; and it causes severe dislocation in their lives [2]. Hemimaxillectomy of acquired palatal defects may predispose the patient to hypernasal speech, leakage of fluids into the nasal cavity, impaired masticatory function, and swallowing difficulties leading to a detrimental impact on the quality of life [3, 4]. Sequentially, it can also affect individual character and trust in social life, leading to social phobia and anxiety [3, 5]. This tends to occur more often in patients with a maxillofacial defect than those with other defects of other organs of the body [3]. Cheeks impart greatly to facial aesthetics. Slumped or hollow cheeks can add years to a person's age. At times, the flanges of the prosthesis can be enough to provide fullness to the cheeks and lips, but sometimes, this is not entirely satisfactory [6].

Prosthodontic rehabilitation of a patient is no longer confined to the replacement of missing teeth alone. The importance of facial aesthetics has become an indispensable piece of dental treatment, and patients are increasingly calling for an improvement in aesthetics. A prosthodontist plays an integral part in restoring the losses in the patient with sunken faces. Sometimes, conventional dentures can fulfill these demands. But, in some instances, extra support to the dentures could be supplied. This can be achieved by using cheek plumpers or cheek lifting appliances [7]. The use of plumper prosthesis in maxillofacial prosthodontics is also well documented [8]. Cheek plumper or cheek lifting appliance is a prosthesis for supporting and lifting the cheek to provide required aesthetics that will increase the self-esteem of the patient [6]. A cheek plumper can be of two types: detachable or undetachable. Undetachable cheek plumpers are conventional single-unit prostheses with extensions on either side of the polished buccal surfaces of the denture. Detachable cheek plumpers are those which can be detached from the denture. This can be achieved by magnets or customized attachments [8].

This article presents prosthodontic management of a young male patient with deformation of the entire right half of the face due to surgical removal of odontogenic myxoma. It describes a method where the clinician utilized a simple, noninvasive, and cost-effective technique to cut the weight of the obturator and to attain aesthetics, utilizing a detachable lip and cheek plumper.

## 2. Case Presentation

**Figure 1:**
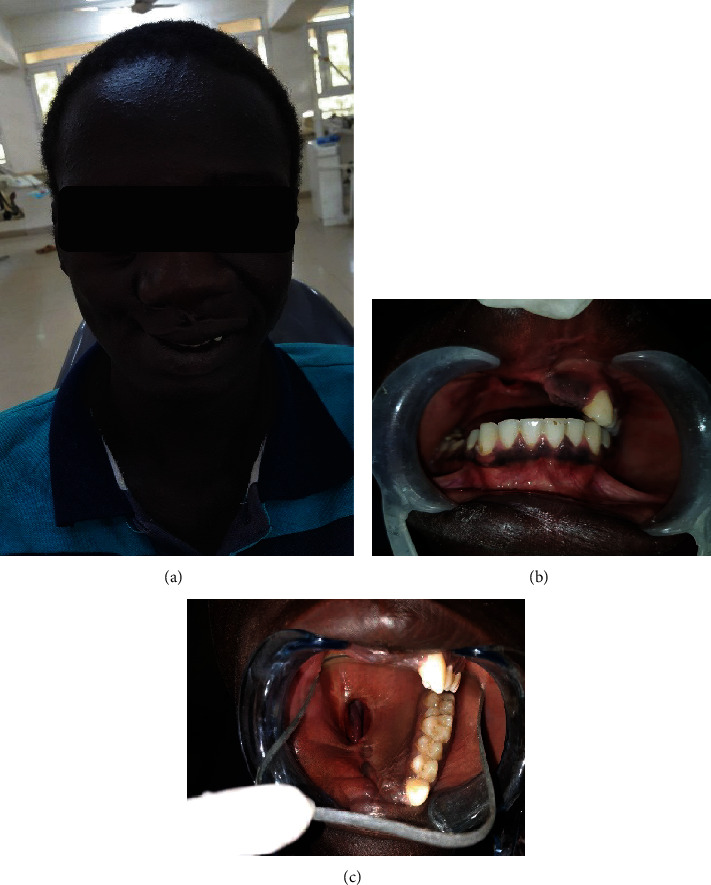
(a) Extra oral view showing facial deformation of the right side; (b) maxillary and mandibular arch occlusion; (c) intraoral view of the maxilla showing defect.

A 19-year-old male attended the Prosthodontic Clinics at Faculty of Dentistry, the University of Khartoum, eight months after the surgical removal of a tumor. The chief complaint was difficulty eating or drinking. Furthermore, he dropped out of school because of his facial deformity and his indistinct voice. Medical and dental history revealed surgical resection of the entire right half of the maxillary alveolar bone due to myxoma. Extra oral examination revealed an asymmetrical face with the loss of lip support and sunken right cheek, depression of the right nasolabial fold, and ptosis on the right eye with many wrinkles around it (Figure 1(a)).

Intraoral examination revealed a well-healed surgical defect in the maxillary right buccal vestibule creating an oroantral communication. The missing teeth were 11, 12, 21, 22, 23, 24, 25, 26, and 27. All the remaining maxillary teeth and complete mandibular dentition were examined clinically as well as radiographically and found to be caries-free with no significant gingival/periodontal problems. Masticatory and phonetic functions of the patient were severely affected due to missing maxillary structures. The panoramic radiograph showed a large radiolucency in the right maxillary region. The defect area extended from the right side, reaching the premaxilla on the left side, crossing the midline, and the patient was classified as class IV according to Aramany's classification of acquired hard palatal defects. The patient had not used an interim obturator (Figures 1(b) and 1(c)).

It was noted that the patient was socially demoralized due to poor aesthetics. He was conscious of his sunken cheek and lip and desired a prosthesis which would make his face look fuller and healthier. A treatment plan was formulated, keeping the patient's demand in mind. The decision was made to treat the patient with a maxillary obturator that would gain its support from the remaining teeth and tissues with a detachable cheek and lip plumper. The use of dental magnets as a means of attachment was elected.

### 2.1. Treatment Procedures

**Figure 2:**
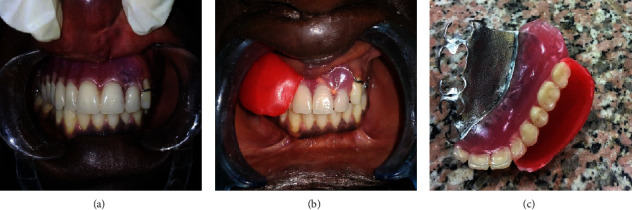
(a) Definitive obturator in maximum intercuspal position; (b) intraoral wax try-in of the plumper; (c) plumper impression hallowed.

Preliminary impressions of both maxillary and mandibular arches were made using irreversible hydrocolloid material (Alginate, Dentsply) for the maxillary and mandibular arches and then poured with type III dental stone. The maxillary study cast was surveyed, and undercut areas were blocked, and an interim obturator was constructed (®DPI Heat Cure) with no teeth incorporation, and wire clasps for retention were attached to it around two selected teeth. The interim obturator was worn by the patient while the definitive obturator was in process. On that study cast, the framework was designed and drawn, and then, a custom tray was constructed using an autocure acrylic resin. Necessary mouth preparations were carried out, and the rest seats were prepared on 12, 13, 14, 15, 16, and 17. Border molding was performed using a high fusing compound (Hiflex Tracing Sticks) for the borders and a low fusing compound (Hiflex impression compound) for the defect area. The final impression was recorded using light-body addition silicone material (Aquasil, Dentsply). This impression was poured with die stone (type IV). After preparation, the master cast was duplicated in refractory material (Whip Mix Beauty-Cast Investment/Gypsum-Bonded) and the partial framework of the cast was fabricated with the help of appropriate wax patterns. The framework was invested and casted using cobalt-chromium alloy (Remanium® GM 380+), and try-in was done with the help of pressure indicator paste (PIP, Mizzy Inc. USA) to affirm the accuracy of fit of the framework. An occlusal bite block was constructed on the framework, and jaw relation was recorded. The casts were mounted; tooth arrangement and a try-in were done. The prosthesis was cured with heat-cured acrylic resin and adequately finished and polished (Figure 2(a)). A pressure indicator paste (PIP) was used to check and eliminate any pressure areas at the insertion appointment. The patient was educated regarding oral hygiene and the future care of the prosthetic device. A recall system for follow-ups was established.

**Figure 3:**
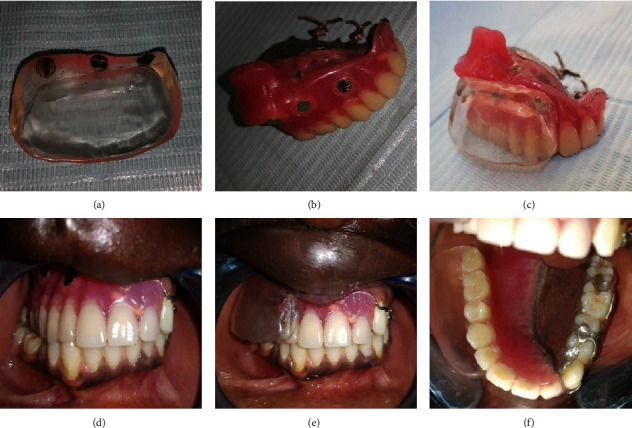
(a) Magnets attached to the removable part of the plumper; (b) magnets attached to the definitive obturator; (c) definitive obturator with plumper parts attached together, (d) intraoral view showing magnets attached to the definitive obturator; (e) intraoral view of the plumper; (f) occlusal view of the view of the plumper.

**Figure 4:**
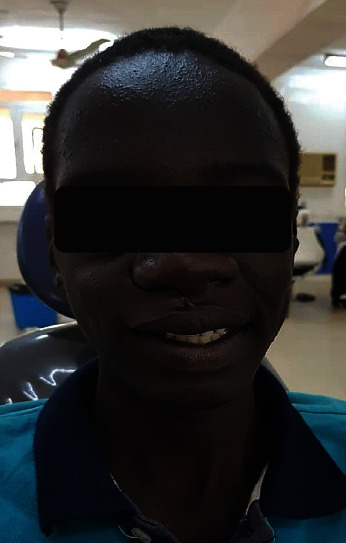
Postoperative frontal view of the patient wearing the lip and cheek plumper having and having better fullness.

One month later, the patient was recalled for the construction of a cheek and lip plumper to give him a fuller upper lip and cheek and asymmetrical facial appearance. Wax was added on the buccal side of the maxillary obturator (Figure 2(b)), and border movements were done until the wax was well adapted. Movements were repeated till the cheek and lip gained the required fullness, and the facial contour was restored, and the patient approved a pleasing appearance. The waxed-up plumper was sent to the dental laboratory which was kept in its position by an index of an addition silicone (Panasil Putty Soft, Kettenbach GmbH & Co. KG, Eschenburg, Germany). The plumper was hollowed (Figure 2(c)), and only a thin border was held back. The waxed-up plumper was separated from the obturator, and three holes were made on the right obturator flange and also on the corresponding area on the plumper. Flasking and dewaxing of the plumper with both parts of the magnets (cobalt-samarium, Ambika Corporation) attached were done (Figure 3(a)). The resultant mold space was then packed with a clear heat-activated denture base resin, and curing procedures were completed (Figure 3(b)). After curing, the magnets of the obturator were attached (Figure 3(c)). Trimming, finishing, and polishing procedures were performed (Figures 3(d)–3(f)). The patient was given instructions on the use of the plumper and was encouraged to make efforts to learn to adapt to it. Within one week, the patient expressed satisfaction in his aesthetic (Figure 4) and even signed up for school.

## 3. Discussion and Conclusion

Facial aesthetics plays a vital role in shaping the psychology of patients. The primary function of a maxillofacial prosthesis is to restore the masticatory function, speech, mastication, and aesthetics. Maxillofacial disfigurement can be congenital, developmental, traumatic, or because of ablative surgery. Such defects compromise appearance and function and render an individual incapable of leading a relatively healthy life and affect his\her psyche. As the patient's quality of life gets worse, social integration becomes difficult, and the expectation of returning to “normalcy” collapses. The prognosis for a successful treatment result depends on getting a correct diagnosis and anticipating issues beyond the land of dentistry alone [9].

The established method of treating a sunken cheek is placing via an additional row of teeth to achieve the fullness of the face [10]. However, this can affect both the retention and stability of the obturator and also cause muscle fatigue. Cheek plumper is a prosthetic device used to support and flesh out the severely sunken cheeks [6]. They were previously used as intraoral splints in patients with Bell's palsy [11]. Such prosthesis is indicated in patients with maxillofacial defects or for restoring aesthetics in patients with hollow or sunken cheeks [12]. Although there are various attachments available for the fabrication of detachable cheek plumpers such as magnets, push buttons, and orthodontic separators, each has its pros and cons [10]. Dental magnets have been used in this case, and they have the benefit of being small and easy to insert because of their magnetic forces and are comfortable to remove and clean. Nevertheless, over a point of time, the magnets used intraorally require replacement due to a lack of long-term durability in oral conditions. The patient was informed about the limitations, and he was put in a six-month recall for a regular checkup and to replace the magnets if required. Conventional cheek plumpers, when used, append to the weightiness of the prosthesis, which can cause muscle fatigue and denture instability [13].

This case report described a secure method to restore facial form by fabrication of detachable plumper and the use of dental magnets to improve the facial aesthetics in a patient with extremely sunken cheeks and lips without affecting the retention of the obturator. In this case, extra lightweight plumper was used for compensation of severely slumped cheeks and lips, with minimal additional weight to the obturator, despite the massive level of compensation required. Assessing the tolerable width for the plumper using scientific and physiological knowledge has not been reported until now. The magnet-retained plumper prosthesis successfully restored the contour of the cheek and improved the aesthetics and the psychological well-being of the patient, and his lost smile was given back. Management of a patient with a maxillary defect should not be confined to the restoration of oral functions only but should equally include the identification of the patient psychological needs and appearance concerns.

This article has described a simple, noninvasive, and cost-effective method to improve facial appearance in patients with hollow faces. An effort was made to improve a patient's appearance by providing better support. The plumper prosthesis successfully improved the patient's appearance to the extent that comfort and function would permit and encourage self-esteem.
